# Facteurs pronostiques de la mortalité néonatale au CHU Sourô Sanou de Bobo-Dioulasso, Burkina Faso: une étude transversale

**DOI:** 10.11604/pamj.2024.47.154.36138

**Published:** 2024-04-02

**Authors:** Cheick Ahmed Ouattara, Rasmatou Tinto, Isidore Tiandiogo Traore, Seydou Traore, Hassane Tamboura, Joseph Bamouni, Ziemlé Clément Meda, Léon Gueswende Blaise Savadogo

**Affiliations:** 1 Université Nazi Boni, Bobo-Dioulasso, Burkina Faso; 2 Centre Hospitalier Universitaire de Souro Sanou, Bobo-Dioulasso, Burkina Faso

**Keywords:** Mortalité, nouveau-né, pronostic, Afrique, Mortality, newborn, prognosis, Africa

## Abstract

**Introduction:**

afin de contribuer à la réduction de la mortalité néonatale au Burkina Faso, nous avons identifié les facteurs pronostiques de la mortalité néonatale au Centre Hospitalier Universitaire Sourô Sanou.

**Méthodes:**

il s'est agi d'une étude transversale et analytique couvrant la période du 25 juillet 2019 au 25 juin 2020 qui s'est déroulée dans le service de Néonatalogie. Elle a consisté à une revue des dossiers de patients, des registres de consultations et d´hospitalisation. Les facteurs pronostics de mortalité néonatale ont été identifiés par un modèle de Cox-Snell.

**Résultats:**

les données de 1128 nouveau-nés ont été analysées. La mortalité néonatale était de 29,8%. La plupart de ces décès (89%) étaient survenus en période néonatale précoce. Le poids moyen à l'admission des nouveau-nés était de 2 285,8 ± 878,7 et 43,6% avaient un poids normal. Huit cent vingt et un (821(38,58%)) et 758(35,62%) nouveau-nés avaient été hospitalisés pour une infection et une prématurité respectivement. Le lieu de l'accouchement (HR autre structure= 1,43[1,17-1,74]), le sexe du nouveau-né (HR masculin = 1,29[1,10 - 1,52]), son poids à l´admission (HR poids < 1000g = 5,45[3,81 - 7,79]) et le diagnostic principal (HR asphyxie= 1,64[1,30-2,08]) étaient les facteurs pronostics de mortalité néonatale.

**Conclusion:**

le relèvement des plateau-techniques pour l'investigation étiologique des infections, la prise en charge efficiente des nouveau-nés de faible poids et souffrant de détresse respiratoire permettraient de réduire considérablement la mortalité néonatale intra hospitalière à Bobo-Dioulasso.

## Abstract (en)

**Introduction:**

to help reduce neonatal mortality in Burkina Faso, we identified the prognostic factors for neonatal mortality at the Sourô Sanou University Hospital.

**Methods:**

we conducted a cross-sectional and analytical study in the neonatal department from July 25, 2019 to June 25, 2020. Patients’ medical records, consultation and hospital records were reviewed. Prognostic factors for neonatal mortality were identified using a Cox model.

**Results:**

data from 1128 newborn babies were analysed. Neonatal mortality was 29.8%. Most of these deaths (89%) occurred in the early neonatal period. The mean weight of newborns at the admission was 2,285.8 ± 878.7 and 43.6%. They were at a healthy weight. Four out of five newborns had been hospitalized for infection or prematurity. The place of delivery (HR weight <1000g = 5.45[3.81 -7.79]) and the principal diagnosis (HR asphyxiation= 1.64[1.30-2.08]) were prognostic factors for neonatal mortality.

**Conclusion:**

improving technical facilities for the etiological investigation of infections and an efficient management of low-weight newborns suffering from respiratory distress would considerably reduce in-hospital neonatal mortality in Bobo-Dioulasso.

## Introduction

La mortalité néonatale est un problème majeur de santé qui est pris en compte dans le troisième objectif du développement durable (ODD) qui vise à réduire la mortalité des enfants de moins de cinq ans, et particulièrement celle des nouveau-nés à douze pour mille naissances vivantes en 2030. En effet chaque année, 5,2 millions d´enfants meurent avant l´âge de cinq ans [1]. La moitié de ces décès survient en période néonatale et les pays en développement sont les plus touchés. Au Burkina Faso la mortalité néonatale est estimée à près de 26 pour 1000 naissances vivantes. La ville de Bobo-Dioulasso abrite le CHU Sourô Sanou (CHUSS), centre de référence de quatre des treize régions du pays. Le service de néonatologie du CHUSS est le seul service public de néonatologie de cette aire sanitaire. Des études antérieures y ont été menées et ont rapporté une mortalité néonatale intra hospitalière variant de 25% à 28% [2,3]. Peu d´études ont cependant abordé les déterminants de cette forte mortalité dont la connaissance est indispensable pour la mise en place d´intervention de contrôle de cette mortalité. L'objectif de cette étude est d'identifier les facteurs pronostiques de la mortalité néonatale au service de néonatologie du CHUSS.

## Méthodes

**Cadre et population de l'étude:** le Centre Hospitalier Universitaire Sourô Sanou est situé dans la région des hauts bassins au sud-ouest du Burkina Faso. Il s'agit d'un hôpital national qui se situe au dernier niveau de la pyramide sanitaire du pays. Il constitue principalement le centre sanitaire de référence de quatre des treize régions du Burkina Faso avec environ six millions d'habitants. Il dispose d'un service de néonatologie avec 42 berceaux, 4 couveuses, 2 aspirateurs et de 7 plaques chauffantes. La collecte des données a été réalisée par revue documentaire des dossiers et des registres d'hospitalisation entre le 1^er^ janvier et le 31 juillet 2021 et couvre les données du 25 juillet 2019 au 25 juin 2020. La population étudiée était constituée des nouveau-nés âgés de 0 à 28 jours hospitalisés dans le service de néonatalogie du CHUSS pendant la période d'étude. Tous les nouveau-nés dont les dossiers médicaux ou le registre d´hospitalisation étaient exploitables étaient éligibles.

**Variables:** les variables collectées étaient relatives aux caractéristiques sociodémographiques des mères (âge, situation matrimoniale, niveau d´instruction, milieu de résidence) et des nouveau-nés (âge, sexe, lieu d´accouchement, structure de provenance),aux antécédents anténataux (gestité, parité, nombre de consultations prénatales, statut vaccinal antitétanique, prophylaxie antianémique et antipaludique, pathologies maternelles), périnataux (terme, poids de naissance, mode d'accouchement, type de présentation, complication pendant l'accouchement, anomalie congénitale, réanimation) et postnataux (motif de consultation, diagnostic en hospitalisation, durée de séjour, poids à la sortie et mode de sortie). Le mode de sortie (vivant ou décédé) a été la variable dépendante.

**Sources et collectes des données:** les données ont été collectées à partir des dossiers médicaux et les registres d´hospitalisation dans le service de néonatologie à l'aide d'un questionnaire structuré dans un format électronique (Epi-Info). Le questionnaire a été pré-testé puis validé. La base de données a été stockée dans un ordinateur protégé par un mot de passe au bureau du service. Le nettoyage des données, ainsi que l'analyse, ont été effectués à l’aide du même logiciel R 4.0.1.

**Taille d´échantillon:** avec une précision de ± 5%, un risque alpha = 5% et pas d'effet de conception (Deff = 1), nous avons besoin d'au moins 384 nouveau-nés pour un taux de mortalité attendu de 50%. Nous avons procédé à un échantillonnage exhaustif des nouveau-nés hospitalisés durant la période concernée par l'étude.

**Analyse des données:** les variables avec des données manquantes ont été écartées de l'analyse. Les variables quantitatives ont été décrites par la moyenne et l´écart type et les variables qualitatives par l´effectif ou la proportion. La variable poids du nouveau-né a été transformé en variable qualitative en utilisant les bornes 1000, 1500, 2500 et 4000 décrits dans la littérature et tenant compte de l´effet du poids sur le pronostic néonatal décrit. La méthode de Kaplan Meier a été utilisée pour déterminer la durée de survie et tracer la courbe de survie. Le modèle de Cox-Snell a été utilisé pour déterminer les liens entre la variable d'intérêt, statut du nouveau-né à sa sortie de l´hôpital et les différentes variables explicatives. Après avoir effectué une analyse bivariée à l'aide du modèle de Cox-Snell, nous avons choisi un niveau de signification de 20%, en dessous duquel toutes les variables explicatives associées ont été incluses dans le modèle multivarié. L'inclusion des variables dans le modèle a été déterminée à l'aide d'une procédure pas à pas ascendant et descendant. Le critère d'information d'Akaike a servi de critère de choix du meilleur modèle et l'adéquation du modèle a été testée avec les résidus de Cox-Snell [4].

## Résultats

Les données de 2128 nouveau-nés sont été incluses dans l'analyse. Les données socio-démographiques des mères en dehors du milieu de résidence étaient manquantes dans 54% des cas et n´ont pas été prises en compte dans l´analyse.

**Caractéristiques sociodémographiques:** mille cent quatre-vingt-dix, soit 56% des nouveau-nés étaient de sexe masculin. Leur âge moyen était de 2,5 ± 5,3 jours. Mille huit cent quatre-vingt-un (88%) nouveau-nés ont été admis en période néonatale précoce dont 63% le jour de leur naissance. Les accouchements avaient eu lieu dans 97% des cas dans une formation sanitaire. Les nouveau-nés ont principalement été référés de la maternité du CHUSS (37,2%) et des centres médicaux avec antenne chirurgicale (CMA) (34,8%). Plus de quatre mères de nouveau-nés sur cinq résidaient en zone urbaine (82%).

### Antécédents anténataux et périnataux

Le nombre moyen de grossesses et d'accouchements des mères était respectivement de 2,4 ± 1,9 et de 2,3 ± 1,8 avec une grossesse multiple sur cinq. Plus de la moitié des mères étaient des primigestes (52%) et primipares (53%). Le nombre moyen de consultations prénatales était de 3,4 ± 1,2 consultations. La majorité de ces femmes ont bénéficié d´une prévention antitétanique (93,7%), antipaludique (94,1%) et antianémique (96%). Treize pourcents des mères ont présenté une pathologie au cours de la grossesse et le paludisme (5,1%) était la pathologie la plus rencontrée. Le taux de césarienne a été de 19%. La présentation du sommet était prédominante (98%). Il a été notifié 220 cas, soit 10% de rupture prématurée des membranes et 205 cas soit 7% de complications au cours de l´accouchement (souffrance fœtale (106), la dystocie dynamique (73) et l´hémorragie du post-partum immédiat (26)). Vingt-neuf pourcents des nouveau-nés ont été réanimés.

**Morbidité néonatale:** l´âge gestationnel moyen a été de 34,8 ± 3,8 semaines et 758(35%) des nouveau-nés étaient prématurés. Aucun cas de post-terme n´a été rapporté. Quatre pourcents d´entre eux présentaient une malformation congénitale. Le poids moyen à l´admission des nouveau-nés était de 2 285,8 ± 878,7 et 43,6% avaient un poids d´au moins 2500 g. Sept cent cinquante-huit (35,62%) nouveau-nés avaient été hospitalisés pour une infection. Le poids moyen à la sortie était de 2 231,7 ± 878,2.

### Mortalité intra-hospitalière

**Figure 1:**
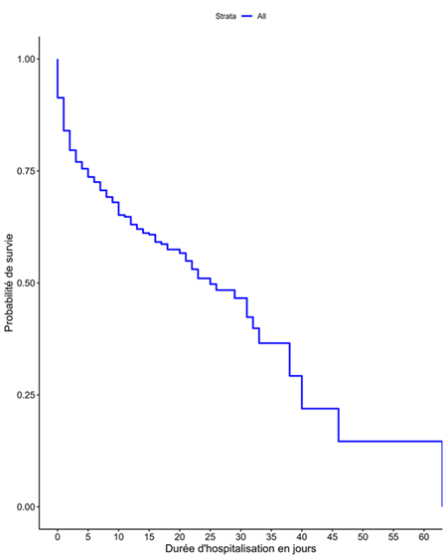
courbe de survie des nouveau-nés admis aux CHU Sourô Sanou, Burkina Faso, 2019-2020

Six cent trente et cinq décès de nouveau-nés sur 2 128 admissions ont été enregistrés soit une mortalité néonatale intra hospitalière de 29,8%. La durée médiane de survie était de 5 jours avec des extrêmes de 0 et 63 jours. La Figure 1 représente la courbe de survie des nouveau-nés (Figure 1). La plupart de ces décès (89%) sont survenus en période néonatale précoce. La mortalité était plus élevée lorsque les nouveau-nés avaient été référés d´une autre structure de santé, 33% par rapport aux références de la maternité du CHUSS, 24%. Vingt et six pourcents des nouveau-nés à terme étaient décédés contre 37% des nouveau-nés prématurés. Les proportions de décès étaient respectivement de 64%, 47%, 22% et 26% pour des termes de moins de 28 semaines, 28 à 31 semaines, 32 à 36 semaines et au moins 37 semaines. En fonction du poids à l'admission, 71/81(88%) nouveau-nés de moins de 1000 g, 189/412(46%) nouveau-nés ayant un poids compris entre 1000 g et 1500 g, 171/696(25%) nouveau-nés ayant un poids compris entre 1500 g et 2500 g, 194/883(22%) nouveau-nés ayant un poids compris entre 2500 g et 4000 g et 10/56(18%) nouveau-nés ayant un poids de plus de 4500 g étaient décédés. La durée moyenne d´hospitalisation était plus élevée chez les nouveau-nés vivants (7 jours) que chez les nouveau-nés décédés (3,3 jours).

### Facteurs pronostiques

A l´analyse bivariée, le nombre de consultation prénatale, la survenue d'une pathologie au cours de la grossesse, la prise de sulfadoxine pyriméthamine, le terme de la grossesse à l´accouchement, le lieu de l´accouchement, le mode d´accouchement, le sexe du nouveau-né, son âge, son poids, la structure de santé l´ayant référé et le diagnostic principal était significativement associés à la mortalité néonatale au seuil de 20%. Le modèle de survie avec l´ensemble de ces variables avait un critère d´information d´Akaike (AIC) de 8945,23. Le meilleur modèle pronostique associait la survenue d´une pathologie au cours de la grossesse, le lieu d´accouchement, le sexe, le poids à l´admission et le diagnostic en hospitalisation avec un AIC de 8925,95 (Tableau 1). Les nouveau-nés référés d´autres formations sanitaires avaient 1,43 fois plus de risque de décès que les nouveau-nés au CHU Sourô Sanou (HR= 1,43; IC95%= 1,17 - 1,74).

**Tableau 1:** facteurs pronostiques de décès néonatal au CHU Sourô Sanou, Burkina Faso, 2019-2020

		HRa brut	95% ICb	p-value	HR ajusté	95% IC	p-value
Sexe	Masculin	1,20	1,03 ; 1,41	0,022	1,30	1,10 ; 1,52	0,002
Résidence	Rural	0,99	0,81 ; 1,22	0,94			
Diagnostic	Infection						
	Asphyxie	1,52	1,21 ; 1,92	<0,001	1,64	1,30 ; 2,08	<0,001
	Prématurité	1,61	1,33 ; 1,93	<0,001	0,96	0,75 ; 1,23	0,7
	Autre	0,86	0,59 ; 1,25	0,43	0,84	0,57 ; 1,22	0,4
Supplémentation en fer	Non	1,13	0,79 ; 1,62	0,50			
Lieu d'accouchement	CHUSS						
	Autre centre	1,47	1,21 ; 1,79	<0,001	1,43	1,17 ; 1,74	<0,001
	Domicile	1,26	0,78 ; 2,05	0,34	0,86	0,52 ; 1,42	0,6
Mode d'accouchement	Césarienne	0,74	0,59 ; 0,93	0,009			
Présentation	Autre	1,27	0,76 ; 2,12	0,37			
Grossesse multiple	Non	0,87	0,72 ; 1,04	0,13			
Rupture prématuree des membranes	Oui	1,00	0,77 ; 1,28	0,98			
Réanimation	Oui	1,10	0,93 ; 1,31	0,26			
A terme	Non	1,30	1,11 ; 1,52	0,001			
Période néonatale	Tardif	0,84	0,65 ; 1,08	0,17			
Poids(g)	Normal						
	<1000	4,22	3,21 ; 5,57	<0,001	5,45	3,81 ; 7,79	<0,001
	<1500	1,72	1,40 ; 2,11	<0,001	2,21	1,66 ; 2,94	<0,001
	<2500	1,04	0,85 ; 1,28	0,70	1,23	0,98 ; 1,55	0,072
	>4000	0,86	0,46 ; 1,63	0,65	0,89	0,47 ; 1,70	0,7
Age		0,98	0.97, 1.00	0.050			
Nombre de consultation prénatale		0,88	0,83 ; 0,94	<0,001			
Gestité		1,01	0,97 ; 1,05	0,66			
Parité		1,00	0,96 ; 1,04	0,89			

Les nouveau-nés de sexe masculin étaient 1,29 fois plus à risque de décès que les nouveau-nés de sexe féminin (HR= 1,29; IC95%=1,10 - 1,52). Les nouveau-nés admis avec un poids inférieur à 1000 g avaient 5,45 fois plus de risque de décès (HR= 5,45; IC95%=3,81 - 7,79) que ceux admis avec un poids normal (2500 à 4000 g). Les nouveau-nés admis avec un poids inférieur à 1500 g avaient quant à eux, 2,21 fois plus de risque de décès (HR= 2,21; IC95%= 1,66 - 2,94). Le diagnostic principal asphyxie était 1,64 (HR= 1,64; IC95%=1,30 - 2,08) fois plus associée aux décès par rapport au diagnostic principal infection néonatale. Il n´y avait pas de différence de risque entre le diagnostic principal infection néonatale et les autres diagnostics.

## Discussion

### Mortalité intra hospitalière

La mortalité néonatale dans notre étude (29,8%) était similaire à celle de 27,9% rapporté par Barro M *et al*. [2] dans le même hôpital entre 2010 et 2016. Ce taux élevé, persistant pourrait s´expliquer par la situation économique et le faible niveau de développement du système sanitaire en général et néonatal en particulier au Burkina Faso comme en témoignent les taux élevés rapportés dans d´autre pays à ressources limitées [5-8] qui contraste avec les taux rapportés dans les pays développés [9]. En effet malgré que le service de néonatologie du CHU Sourô Sanou soit le seul service public de néonatalogie qui couvre quatre des treize régions sanitaires du Burkina Faso.

### Facteurs pronostiques

Dans cette étude, les facteurs pronostiques de mortalité néonatale étaient le lieu de l´accouchement, le sexe du nouveau-né, son poids à l´admission et le diagnostic principal. Les nouveau-nés venant d´autres formations sanitaires avaient un plus mauvais pronostic comparativement à ceux qui étaient nés à la maternité du CHU Sourô Sanou. Le retard lié à la décision de transfert et au trajet à parcourir pour atteindre le CHUSS pourrait expliquer cette différence [10]. La prédominance masculine trouvée dans notre étude (56%) a également été rapportée dans les études Barro M *et al*. [2] au Burkina Faso, de Yenan *et al*. en Côte d´Ivoire [11], Sylla *et al*. au Mali [12].

Plusieurs études ont identifié le poids du nouveau-né comme un facteur prédictif de la mortalité néonatale [2,5,8,13-17]. En effet, une grande part des nouveau-nés de faibles poids sont prématurés ou issue d´un retard de croissance intra utérine avec un système immunitaire et de nombreuses fonctions immatures donc plus fragiles. Leur prise en charge requiert un plateau technique et des moyens financiers qui ne sont pas toujours réunis dans nos pays. Cela pourrait expliquer le risque de mortalité élevée chez les nouveau-nés de moins de 1500 g. Les principaux diagnostics en hospitalisation sont les mêmes qui ont été retrouvés dans plusieurs études en Afrique subsaharienne [2,14-17]. Ils sont dominés par l´infection néonatale dont l´investigation étiologique est presqu´inexistante et la prise en charge basée sur une antibiothérapie empirique [18]. La mortalité néonatale élevée et l´émergence des bactéries résistantes constituent des arguments pour évaluer l´efficacité de cette antibiothérapie et l’adaptée si nécessaire. Cela pourrait contribuer à une réduction de la mortalité néonatale. Seule l’asphyxie constituait un surplus de risque de mortalité néonatale par rapport à l´infection. Le développement de stratégies pré hospitalières, une amélioration du plateau technique de prise en charge, de surveillance et le renforcement des compétences en matière de réanimation du nouveau-né pourrait améliorer le pronostic des nouveau-nés atteints de détresse respiratoire.

Les principales limites à notre étude sont méthodologiques. Les études transversales ne permettent pas d´établir une association causale entre la variable dépendante et les variables indépendantes. Aussi l'utilisation des données rétrospectives réduit les variables d´ajustement à celles qui sont disponibles. Ainsi certains dossiers médicaux étaient inexploitables et nous n´avons pu utiliser certaines variables maternelles d´intérêt en rapport avec les mères car non renseignés. En outre bien que les résultats soient généralisables à la population du bassin de couverture de l´hôpital, il s'agit de données hospitalières dont la validité externe peut être influencée par le recours aux soins.

## Conclusion

La mortalité néonatale reste élevée au CHU Sourô Sanou avec plus d'un décès de nouveau-né sur quatre admis. La principale pathologie du nouveau-né est l'infection. Les facteurs de mauvais pronostic sont la naissance hors de la maternité du CHU Sourô Sanou, le sexe masculin, le faible poids et l'asphyxie. Le relèvement des plateau-techniques et des compétences dans les formations sanitaires primaires, et dans les hôpitaux pour l'investigation étiologique des infections, la prise en charge des nouveau-nés de faible poids et souffrant de détresse respiratoire permettrait de réduire considérablement la mortalité néonatale intra hospitalière à Bobo-Dioulasso.

### Etat des connaissances sur le sujet

- *La mortalité néonatale intra hospitalière reste élevée dans les pays en voie de développement;*
- *Les principaux facteurs associés à cette mortalité sont le faible poids de naissance et les infections*.

### Contribution de notre étude à la connaissance

- *Notre étude met en évidence que même si l'infection néonatale demeure la principale cause d'admission de nouveau-nés à l'hôpital Sourô Sanou, d´autres pathologies comme l'asphyxie néonatale sont associées à un risque de mortalité plus élevée;*
- *Aussi cette étude montre que les facteurs pré hospitaliers doivent être pris en compte dans l'élaboration de stratégie de réduction de la mortalité néonatale*.
